# Reducing Time to Antibiotic Delivery in Pediatric Patients with Suspected Sepsis

**DOI:** 10.1097/pq9.0000000000000516

**Published:** 2021-12-16

**Authors:** Vikram K. Raghu, Meghan Gill, Elizabeth Ferguson, Adrienne Marcinick, Gabriella Butler, Christopher Horvat, Kelli Crowley

**Affiliations:** From the * Department of Pediatrics, UPMC Children’s Hospital of Pittsburgh, Pittsburgh, Pa.; † Department of Pharmacy, UPMC Children’s Hospital of Pittsburgh, Pittsburgh, Pa.; ‡ Division of Health Informatics, UPMC Children’s Hospital of Pittsburgh, Pittsburgh, Pa.; § Department of Critical Care Medicine at UPMC Children’s Hospital of Pittsburgh, Pittsburgh, Pa.

## Abstract

The Children’s Hospital Association’s Improving Pediatric Sepsis Outcomes (IPSO) collaborative is a multi-center quality improvement (QI) learning collaborative of 61 U.S. children’s hospitals that seeks to improve sepsis outcomes through collaborative learning and reliable implementation of evidence-based interventions in pediatric emergency departments, intensive care units, general care units, and hematology/oncology units. Specifically, IPSO’s goals are to decrease sepsis-attributable mortality and prevent hospital-onset sepsis among children.

The following 10 abstracts represent a select group of projects undertaken by IPSO participating hospitals that were presented at one of three collaborative events in 2020 and 2021. IPSO’s Research Workgroup reviewed all submitted abstracts and selected the top 10 for inclusion in this Supplement

## Background:

Timely antibiotic administration reduces sepsis-related morbidity and mortality. The pharmacy department plays a critical role in timely antibiotic dispensing, thereby improving pediatric sepsis outcomes.

## Objectives:

We aimed to decrease the time from antibiotic order to delivery in suspected pediatric sepsis.

## Methods:

We used a Lean/Six Sigma framework to improve antibiotic timing. Stakeholders included clinical pharmacists, pharmacy staff, nurses, physicians, and medication delivery assistants. We created a process map, which helped identify three key steps in the process: (1) use of a sepsis order set, (2) nurse-to-pharmacy phone communication, and (3) delivery by pneumatic tube. We performed educational interventions for pharmacy, nursing, and house staff. We reviewed all antibiotic orders placed following a positive sepsis screen for acute care patients at UPMC Children’s Hospital of Pittsburgh between 4/1/2019 and 10/31/2019, which spanned before and after our interventions. The primary outcome measure was time from antibiotic order to delivery.

## Results:

We reviewed 94 antibiotic orders for 64 patients. Following interventions, a statistically significant reduction in orders delivered <45 minutes after placement was observed (*P* = 0.015). Median time to delivery was significantly shorter for “Stat” orders (42.5 minutes; IQR: 20, 48) than “routine” orders (51 minutes; IQR: 41, 65; *P* < 0.005). The median time to administration when delivered by pneumatic tube was significantly less than when delivered by hand to a healthcare provider (53 [IQR 41, 48] versus 79 [IQR 61, 108] minutes; or to the automated dispensing cabinet (90 [IQR 63, 122] minutes; *P* < 0.0005).

## Conclusion:

Interventions driven by the pharmacy department facilitate improved delivery time for pediatric sepsis antibiotic orders.

